# Computation and Communication Evaluation of an Authentication Mechanism for Time-Triggered Networked Control Systems

**DOI:** 10.3390/s16081166

**Published:** 2016-07-25

**Authors:** Goncalo Martins, Arul Moondra, Abhishek Dubey, Anirban Bhattacharjee, Xenofon D. Koutsoukos

**Affiliations:** 1 Department of Electrical and Computer Engineering, University of Denver, Denver, CO 80208, USA; Goncalo.Martins@du.edu; 2 Institute for Software Integrated Systems (ISIS), Department of Electrical Engineering and Computer Science, Vanderbilt University, Nashville, TN 37212, USA; Arul.Moondra@vanderbilt.edu (A.M.); Abhishek.Dubey@vanderbilt.edu (A.D.); Anirban.Bhattacharjee@vanderbilt.edu (A.B.)

**Keywords:** time-trigger architectures, wireless TTA, secure messages, cyber-physical systems, timing and performance analysis, HMAC

## Abstract

In modern networked control applications, confidentiality and integrity are important features to address in order to prevent against attacks. Moreover, network control systems are a fundamental part of the communication components of current cyber-physical systems (e.g., automotive communications). Many networked control systems employ Time-Triggered (TT) architectures that provide mechanisms enabling the exchange of precise and synchronous messages. TT systems have computation and communication constraints, and with the aim to enable secure communications in the network, it is important to evaluate the computational and communication overhead of implementing secure communication mechanisms. This paper presents a comprehensive analysis and evaluation of the effects of adding a Hash-based Message Authentication (HMAC) to TT networked control systems. The contributions of the paper include (1) the analysis and experimental validation of the communication overhead, as well as a scalability analysis that utilizes the experimental result for both wired and wireless platforms and (2) an experimental evaluation of the computational overhead of HMAC based on a kernel-level Linux implementation. An automotive application is used as an example, and the results show that it is feasible to implement a secure communication mechanism without interfering with the existing automotive controller execution times. The methods and results of the paper can be used for evaluating the performance impact of security mechanisms and, thus, for the design of secure wired and wireless TT networked control systems.

## 1. Introduction

There is a continuing demand to provide services required for predictable message communication of safety-critical control applications, such as drive-by-wire in automotive systems. In such systems where the failure of the system can lead to serious injury or even death, it is important that the system provides real-time communication guarantees. For those ultra-dependable systems, the failure rates should be in the order of $10^{-9}$ failures/h [1]. Moreover, networks in automotive systems and avionics require distributed architectures to support safety-critical real-time control. For such systems, Time-Triggered Architectures (TTA) offer significant advantages in terms of safety, reliability and fault tolerance [2,3].

TTA provides a framework that allows the design of distributed, embedded and real-time systems ensuring high-dependability. In particular, TTA provides mechanisms that enable the exchange of precise and synchronous messages and helps to engineer fault-tolerant systems for both control software and networked data communications [4]. Time-triggered networks are beneficial in many applications that include safety-critical systems, especially in-vehicle networks, by managing the complexity of fault-tolerance and analytic dependability models and ensuring highly reliable and deterministic systems [4].

Safety, reliability and fault-tolerance properties have been the main focus of TTA systems. Another important property is to ensure communication security [5,6]. Providing mechanisms to enable secure communications is important to prevent actions by attackers. Typically, TT systems cannot be equipped with traditional processors. Instead, embedded processors are used. Adding security mechanisms may incur significant computational and communication overhead and jeopardize temporal properties. Therefore, it is important to evaluate the computational and communication overhead of implementing such secure communication mechanisms.

The objective of this paper is to perform a comprehensive evaluation of the computational and communication overhead due to message integrity and authentication for TT networked control systems. To meet these security goals, a Hash-based Authentication Code (HMAC) or digital signatures can be used. Digital signatures are normally slower than HMAC methods, but both are capable of providing identical levels of security. The main difference relies on the management of keys and includes or does not the non-repudiation security property, which it is not covered by this work. A fast message integrity and authentication method is preferred due to the fact that TT control systems have restricted timing constraints. In this work, HMAC is implemented and tested to achieve the desired security goals. The first contribution of this work is the analysis and experimental validation of the communication overhead of HMAC for both wired and wireless platforms. In addition, a scalability analysis of the communication overhead with the network size is presented utilizing the experimental results. The second contribution is the experimental evaluation of the computational overhead of HMAC. The implementation is performed using the Linux cryptographic libraries at the kernel level of the selected platforms.

The comprehensive evaluation results presented in the paper show that it is possible to implement HMAC in TT networked control systems and to analyze both the computational and communication overhead. As an example, an automotive application is used, and the results show that it is feasible to implement a secure communication mechanism without interfering with the existing automotive controller execution times. It is shown that for the selected platforms, the overhead is relatively small and does not interfere with the time-triggered control execution and network schedule. Further, the methods and results of the paper can be used for evaluating the performance impact of security mechanisms, and thus, for the design of secure wired and wireless TT networked control systems.

This paper is organized as follows. Section 2 describes the related work. Section 3 formulates the problem and provides a description of the TT systems under evaluation. In Section 5, the theoretical analysis for the communication overhead is presented. In Section 6 and Section 7, the practical evaluation results for the computational and communication overhead are presented, respectively. Section 8 presents the scalability analysis. At last, Section 9 provides the conclusion.

## 2. Related Work

As software complexity increases in networked control systems, it is almost impossible to avoid security flaws. Drive-by-wire systems can be developed based on time-triggered architectures, such as TTEthernet [7]. The authors in [8] discuss security threats that are currently not covered by TTEthernet. A relevant threat pointed out in this work that still triggers attention from the research community is dataflow threats. From a network perspective dataflow threats are related to the way an attacker may modify messages communicated in the network (message modification). The author highlights that in current TTEthernet systems, depending on the location of the internal attack, the attacker can forge all messages that are white-listed at a given port.

For wireless time-triggered architectures, WirelessHART is the first open wireless communication standard specifically designed for process measurement and control applications [9]. Other wireless technologies relying on ZigBee or Bluetooth communications have been used before for TT systems [6]. However, these technologies may not meet the demanding timing requirements of industrial control. The authors in [10] present a comparison study of WirelessHART and ZigBee for industrial applications. Security in communications is addressed and included in WirelessHART, although it is not clear what is the impact on the computational or network overhead by implementing such security measures.

The National Highway Traffic Safety Administration (NHTSA) reported cases about infiltration in automotive control systems and installing malware remotely, using Bluetooth devices and CD [11]. The authors in [12] conduct an experiment to evaluate the fragility of the underlying system structure of a modern automobile system. The work shows how an attacker is able to infiltrate virtually any Electronic Control Unit (ECU). The attacker has the ability to completely circumvent a broad array of safety-critical systems and manipulate and control the automotive functions by ignoring the driver’s input. Such vulnerabilities highlight the importance in securing messages in vehicular networks.

The authors in [13] present a study of current and future bus systems with respect to their security features. The work states that “a fundamental step to improve automotive bus communication security is the encryption of all vehicular data transmission”. A secure automotive communication based on modern cryptographic mechanisms is proposed; however, the performance impact of such security mechanisms is not evaluated.

In [14], the authors provide a study about the implementation, performance and research challenges for secure vehicle communication systems. One of the challenges relies on the integration of security systems or mechanisms for different platforms used in Vehicular Communications (VC). The study proposes an architecture (entitled SeVeCom) with the aim of implementing and deploying specific aspects, such as flexible integration in existing communication stacks, the use of a hardware security module and secure connections of VC onboard units to in-vehicle bus systems. The study focuses mainly on the analysis of the performance and communication overhead of communication between vehicles. The study addresses the computational implications for the selected secure mechanisms, but it does not cover any communication overhead results for in-vehicle bus systems.

In [15], the authors propose a novel vehicle security architecture that incorporates encrypted messages to enable in-vehicle electronic control units to authenticate and participate in an e-voting computation scheme. This scheme allows one to determine whether or not the system is considered secure to initialize. This work is one more step in the direction of securing in-vehicle communications; however, it does not address the computation or communication overhead of using such a secure architecture scheme.

A comparison of different authentication algorithms to facilitate authentication in time-triggered systems is presented in [16]. The work addresses the computational effort required to compute authentication protocols for embedded systems with scarce computational resources. Moreover, it focuses on analyzing two properties of time-triggered transmission channels (sender authenticity and channel integrity) that are applicable to prove a message’s origins. However, it does not address in detail the communication overhead of using authentication algorithms in time-triggered systems.

## 3. Problem Formulation

With the aim to address the feasibility in securing messages for TT systems, a comprehensive evaluation of the computation and communication overhead of the implementation of an authentication mechanism is presented.

**Figure 1:**
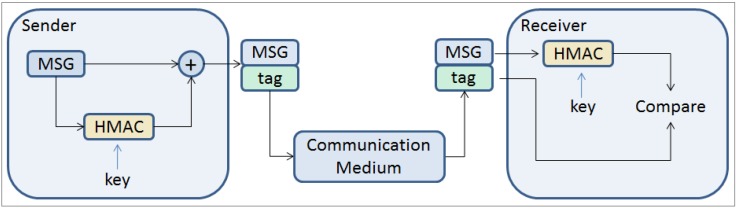
Message authentication scheme.

The general idea is to use a keyed-Hashed Message Authentication Code (HMAC) [17] in order to verify the data integrity and authentication of a message (Figure 1). The first goal is to measure the computational overhead on the sender and receiver nodes due to adding the authentication mechanism. The authentication mechanism generates additional information that needs to be attached to the original message (tag). The second goal is to measure the communication overhead generated on the communication medium as a result of adding information on the message that is desired to be transmitted.

### 3.1. Platforms under Evaluation

The two different TT platforms considered in this evaluation are described below.

#### 3.1.1. Platform A

Platform A is a wired TT network, which represents an automotive communication system. The bus communication system used is TTEthernet (http://www.tttech.com/technologies/ttethernet/). The system is composed of four Electronic Control Units (ECUs) and one TTEthernet switch. The ECUs are self-contained units (IBX-530W box) that include a processor (1.6-GHz Intel Atom processor) with 1 GB of memory and 512 MB of cache, and a real-time operating system based on RTLinux and Ubuntu (Linux Kernel 2.6.24-24-rt). All of the software developed is running in the kernel space managed by the RTLinux scheduler, ensuring real-time execution.

**Figure 2:**
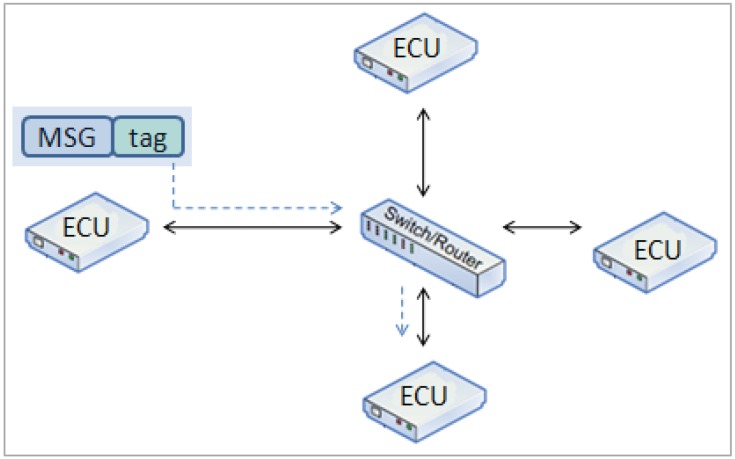
Platform A network topology: star configuration.

The network is a star topology (Figure 2a) that allows communication synchronization among nodes in a centralized way. The nodes are connected through a central switch via bidirectional communication links. Each node communicates with the other nodes by sending messages to the switch, which then relays the messages to the respective receiving nodes. Events occur at a predefined time with precision at the microsecond level. The system uses an off-line scheduling tool that statically creates the bus communication schedule table. This table specifies when messages (e.g., Time-Triggered (TT) or Best Effort (BE) messages) are transmitted by a node and the node that will receive the message. This feature ensures that the network gives priority to TT messages.

#### 3.1.2. Platform B

Platform B is a wireless TT platform, which implements the TT communication scheme in a wireless network. The system includes four identical ARM (Advance RISC Machine) boards. The nodes (or ECUs) are single board units named Trimslice (http://utilite-computer.com/web/models), with a CPU based on the NVIDIA Tegra2 SoC, a dual-core 1-GHz ARM Cortex-A9 CPU with 1 GB of RAM, and it runs an operating system based on Ubuntu 12.04 (Linux Kernel 3.1.10-l4t.r16.02). All of the software developed is running in the kernel space managed by RTLinux scheduler, guaranteeing real-time execution.

**Figure 3:**
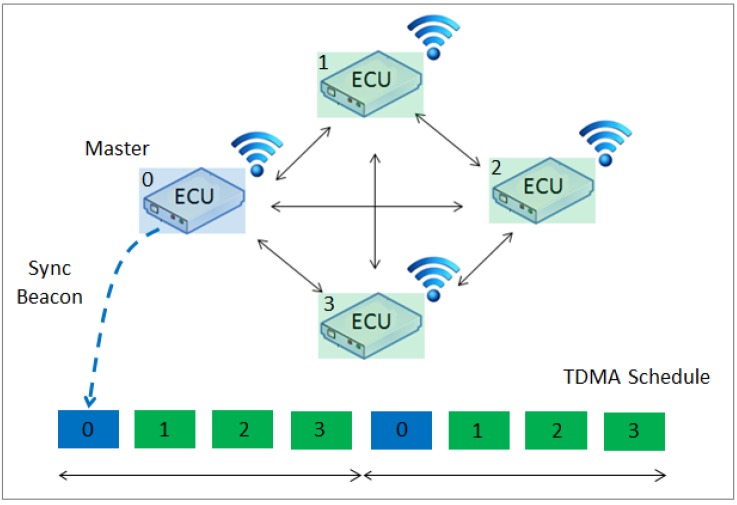
Platform B network topology: mesh configuration.

The nodes are connected in an ad hoc mode (Figure 3) using WiFi 802.11 g at 54 Mbps. To achieve communication synchronization among all of the nodes, a Time-Division Multiplexing Scheme (TDMA) is implemented. Each node has a unique ID number, which corresponds to the respective time slot allocated in the communication scheduler (off-line scheduler). Time synchronization is provided by assigning one node as the master node, which broadcasts a beacon packet in a predefined interval. All of the other nodes adjust their clocks upon receiving the beacon packet and wait until their preassigned time to transmit their data.

### 3.2. Authentication Mechanism

The security goals that we are trying to achieve are message integrity and authentication. Nodes should be authenticated, and the integrity of the messages of the sender’s node should be maintained. The messages should not be able to be tampered with; that is, an attacker should not be able to modify the messages remotely, and the receiver node should only accept messages from authenticated nodes, discarding the other messages.

The technique implemented to authenticate the messages relies on a keyed-Hashed Message Authentication Code (HMAC) [17]. The idea is to generate a tag for the respective message, and then, the tag is appended and transmitted with the original message (Figure 1).

HMAC generates a tag by combining a cryptographic hash function with a secret cryptographic key. The cryptographic hash functions should be one-way and collision resistant. It is computationally infeasible to find a message that corresponds to a given message digest or to find two different messages that produce the same message digest. Any change to a message in transit will, with very high probability, result in a different message digest, and the signature will fail to be verified. The strength of the HMAC depends on the cryptographic strength of the underlying hash function, the size of its hash output and on the size and quality of the key [17].

In this paper, three cryptographic hash functions are implemented and evaluated:

SHA-1: a 160-bit hash function;
SHA-2: SHA-256 hash function with 32-bit words;
SHA-3: Keccak hash function that supports the same hash lengths as SHA-2, but its internal structure is significantly different from the rest of the SHA family [18].

For all of the hash functions described above, a secret cryptographic key with 64 bytes is used. The unique tag message authentication code generated by the hash algorithms simultaneously verifies the data integrity and the authentication of a message. The sender and receiver share the same key.

The message authentication and validation scheme is outlined below:

- The sender node generates a tag for the desired message to transmit;
- The message is appended with the tag, and the combination is transmitted to the receiver node;
- The receiver node separates the message and the tag;
- Then, using the same key as the sender, the receiver node regenerates the tag for the message received;
- The receiver then compares the regenerated tag with the received tag.

The extra message tag overhead in bytes introduced is dependent on the message tag generated by the cryptographic hash-function used in HMAC. For SHA-1, the message tag is 20 bytes, and for SHA-2 and SHA-3, the message tag is 32 bytes.

## 4. Evaluation Metrics

Overhead can be defined as any combination of excess computation time (computational overhead), bandwidth (communication overhead), memory, scheduling (CPU overhead) or other resources that are required to attain a particular goal. The authentication scheme does not have any impact on the CPU overhead. TTA schedulers schedule all of tasks and messages involved in the system offline. This is how TTA guarantees real-time and deterministic networks. The authentication scheme has an impact only on the task execution time level, which is related to the computational overhead. The ECUs used in this work have enough memory to consider memory overhead as negligible. Moreover, memory overhead does not have a direct impact on the network communication system. In this work, the computational overhead and communication overhead impacts of the implemented authentication scheme are considered. The evaluation metric for the computational overhead uses the HMAC execution time.

Regarding the communication performance, time-triggered systems use TDMA to allocate each message a unique access time within a periodic transmission schedule. Based on this technique, the need for an explicit collision-resolution mechanism is eliminated. Each transmitter determines its turn to access the network by checking a time reference. During the design of the communication system the maximum number of nodes that can participate in the TDMA scheduling should be taken into account. Adding an extra node might disturb the correct operation of the already integrated ones [2].

A similar problem is expected to occur if nodes send more information than the maximum allocated per slot. This extra information might be necessary to ensure security during communications. Therefore, it is important to reserve adequate bandwidth in order to ensure schedulability.

**Figure 4:**
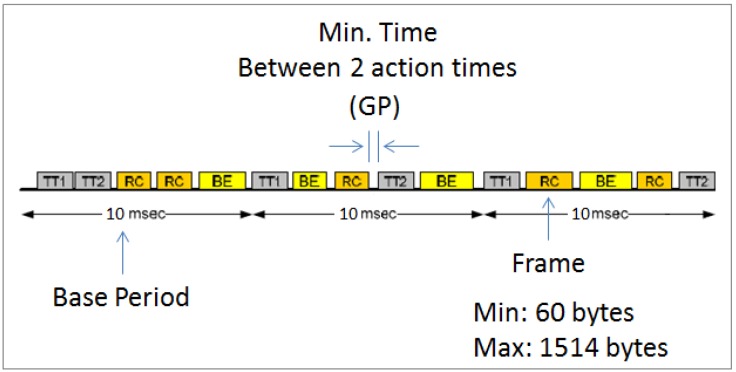
TT TDMA frame.

Figure 4 shows a typical TT TDMA frame. It consists of a Base Period (BP), the minimum time between two action times, also called the Guard Period (GP), and the frame or slot time ($Frame_{time}$).

The maximum number of frames ($NF_{max}$) allowed per BP is the following:

(1) $$NF_{max}=\frac{BP}{(Frame_{time})+GP}$$

where,

(2) $$Frame_{time}=\frac{Packet_{size}}{Transmission_{rate}}$$

Therefore, we select the evaluation metric for the communication overhead to be the maximum number of frames ($NF_{max}$) allowed per BP.

Table 1 summarizes the notation and respective terminology used in this paper.

## 5. Analysis of the Communication Overhead

This section provides the theoretical analysis for the communication overhead by appending the additional information generated by the authentication mechanism to the messages.

### 5.1. Platform A

For Platform A, the theoretical and empirical results assume a 100-Mbits/s TTEthernet switch. The TTEthernet platform defines a minimum frame size of 60 bytes and a maximum frame size of 1514 bytes. It also defines a minimum transmission time between two packets of 0.2 ms (Guard Period (GP) = 0.2 ms). This minimum guard period is used to guarantee that the physical switch has time to route one frame size at its maximum size. Table 2 summarizes the parameters used in Platform A. The Base Period (BP) is 10 ms, and it is selected to ensure that all of the control units will work at the designed and desired rate with the proposed secure mechanism implemented. The maximum packet size used is 60 bytes, which is sufficient for control. As mentioned in Section 3.2, the overhead on the frame size due to the generated hash tag is 20 or 32 bytes (depending on the hash function used).

Table 3 shows the theoretical results for $NF_{max}$ and $Frame_{time}$ with BP = 10 ms and frame sizes of 60 and 80 bytes. It is assumed that GP = 0. The table also shows the theoretical results for $NF_{max}$ and $Frame_{time}$ with BP = 10 ms and the suggested and defined TTEthernet guard period (GP = 0.2 ms).

**Figure 5:**
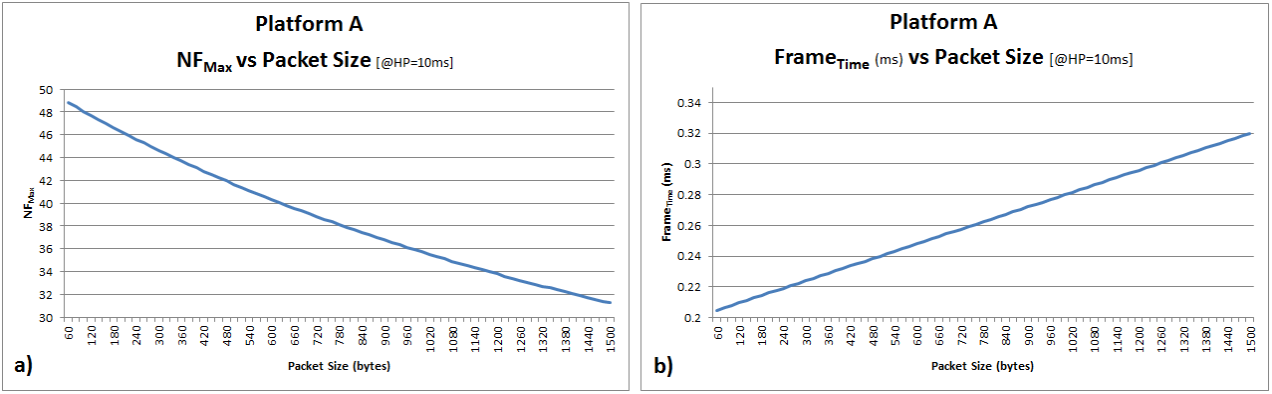
Platform A (**a**) $NF_{max}$ vs. packet size (bytes) with BP = 10 ms and GP = 0.2 ms; (**b**) $Frame_{time}$ (ms) vs. packet size (bytes) with BP = 10 ms and GP = 0.2 ms.

A guard period of 0.2 ms reduces the maximum number of frames per BP drastically. As an example, for the selected guard period and using the message tag overhead introduced by using SHA-1, there is no impact (in theory) on the maximum number of frames by changing the packet size from 60 to 80 bytes (20 bytes message tag overhead). Figure 5a shows the expected impact for $NF_{max}$ per BP by changing the frame size from 60 to 1514 bytes. Figure 5b shows the expected impact for $Frame_{time}$ per BP by changing again the frame size from 60 to 1514 bytes.

### 5.2. Platform B

Platform B has a built-in 802.11 n WiFi card. The maximum data rate is 54 Mbits/s, and the system has the same minimum frame size of 60 bytes. Due to hardware and operating system limitations the maximum frame size is 1300 bytes. It is important to highlight that the guard period or minimum transmission time between two packets is addressed in a different way for Platform B. In the IEEE 802.11 standard, the beginning of each symbol is preceded by a guard interval. The size of the symbol is defined by the type of modulation used during the Orthogonal Frequency-Division Multiplexing (OFDM) encoding process. This guard interval is to guarantee that the receiver has the ability to decode the actual data in time. The guard interval can vary according to the 802.11 standard, and it is included during the calculation of the theoretical maximum data rate.

The guard period for the TDMA analysis has a different value from the 802.11 guard interval. The guard period for the TDMA is added to avoid data loss and to reduce interference, caused by propagation delay. To be consistent with the analysis performed for Platform A, a minimum transmission time between two packets of 0.2 ms (GP = 0.2 ms) is used. Table 4 summarizes the experiment parameters used for this platform.

The Base Period (BP) and the maximum packet size are the same as the ones used for Platform A, BP = 10 ms and 60 bytes, respectively. As mentioned in Section 3.2, the overhead on the frame size due to the generated hash tag is 20 or 32 bytes (depending on the hash function used).

Table 5 shows the theoretical results for $NF_{max}$ and $Frame_{time}$ with BP = 10 ms and changing the frame size from 60 to 80 bytes. The table also shows the theoretical results for GP = 0 (assuming only the guard interval of the Orthogonal Frequency Division Multiplexing (OFDM) encoding process) and for GP equal to the one used for Platform A (GP = 0.2 ms).

Similar to the results from Platform A, with a guard period of 0.2 ms, the maximum number of frames per BP is reduced drastically. The main differences from Platform A are the following: the maximum date rate drops from 100 Mbits/s to 54 Mbits/s; and for GP = 0, there is actually a guard interval inherent to the 802.11 OFDM encoding process that will affect $Frame_{time}$ and, consequently, $NF_{max}$. From Table 5, for the selected guard period and the message tab overhead introduced by using SHA-1, there is no impact (in theory) on the maximum number of frames by changing the packet size from 60 to 80 bytes (20 bytes of message tag overhead).

## 6. Evaluation of the Computational Overhead

This section presents the computational overhead due to implementing the authentication mechanism in the sender and receiver nodes. To ensure deterministic and fast execution, all of the code is implemented at the kernel level on the end nodes.

**Figure 6:**
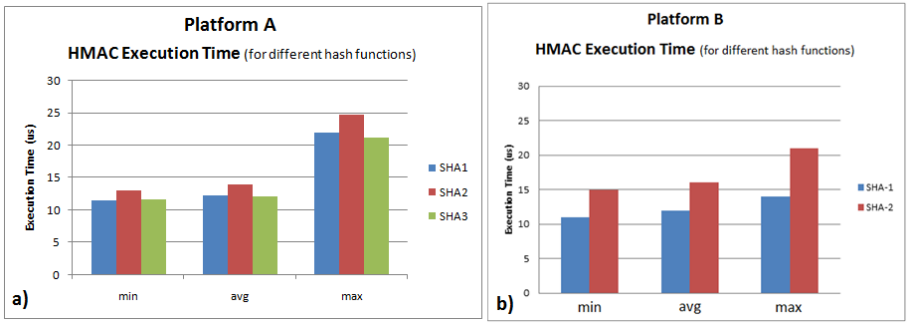
HMAC kernel execution time for different hash functions: (**a**) Platform A; (**b**) Platform B.

In order to evaluate the computational overhead, the HMAC execution time is used as the evaluation metric. The minimum, maximum and average execution time for each hash function (SHA-1, SHA-2 and SHA-3) for a packet size of 60 bytes plus the respective message tag was measured (Figure 6). SHA-3 was not implemented on Platform B due to kernel incompatibilities.

The typical controller code execution time is 1200 μs [19], and for the experiments conducted, the maximum HMAC execution time is 25 μs for Platform A and 20 μs for Platform B. The computational overhead of the implemented hash function is negligible.

## 7. Evaluation of the Communication Overhead

This section presents the experimental analysis of the communication overhead.

**Figure 7:**
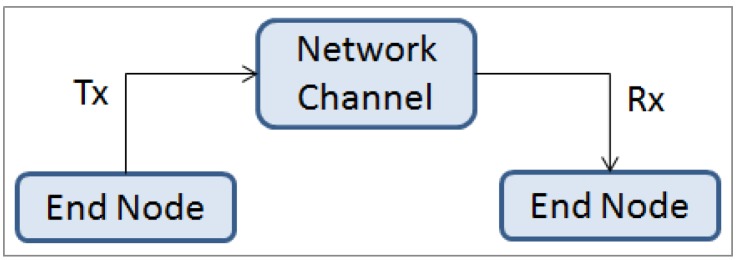
Block diagram for the general physical setup.

The block diagram for the experimental setup is depicted in Figure 7. In order to measure the impact on $NF_{max}$ by adding additional information on the message, it is important to measure the time that a packet takes to travel from one end node to the other through a network channel (wired or wireless). It is desired to measure this interval with as little interference as possible.

### 7.1. Platform A

#### 7.1.1. Physical Measurement Setup

**Figure 8:**
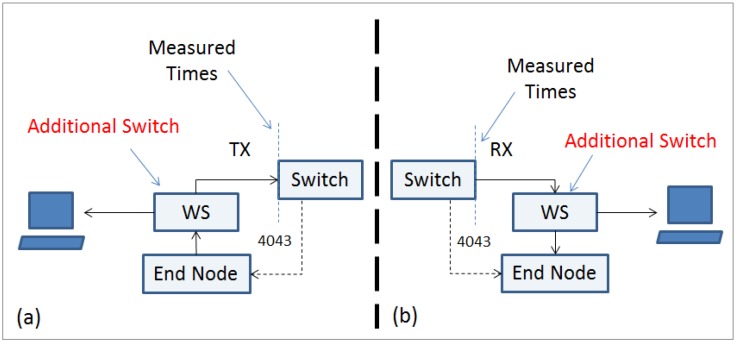
Block diagram for the measurement physical setup: (**a**) Packet arrival at the central switch; (**b**) Packet transmission at the central switch.

For Platform A, the approach relies on “sniffing” the packets on the network channel, in this case, the central switch. The central switch is not configured to broadcast the packets to all ports. In order to monitor the packets during communications, it is necessary to connect a secondary switch that allows the connection of a packet analyzer (e.g., Wireshark http://www.wireshark.org/). Figure 8 shows the block diagram for the measurement setup. The additional switch (Wireshark (WS)) introduces a small delay in the packets’ transmission time, but this delay is consistent and deterministic, as can be seen in Table 6.

The central switch is the master node of the TDMA scheduler. It sends to all nodes a sync beacon at a predefined and deterministic time in a dedicated channel (Channel 4043). This is the reference packet time used for all measurements. Then, the packet arrival time (Tx) and the packet transmission time (Rx) at the switch are measured. With these measurements in conjunction with the sync beacon (Sync Beacons are frames used as time reference) packet timing, it is possible to infer the packet transmission time from the end node to the switch.

#### 7.1.2. Experimental Results

Wireshark is used as the packet analyzer, and it allows the visualization of the sync beacon packets, the packet arrival and packet transmission timings. All of these measurements are related to the central switch, because the central switch is responsible for sending the synchronization beacon. Four tests are conducted in order to evaluate the switch performance at its minimum and maximum frame size. For all of the tests, data are collected for 5 min. The tests are the following:

- Measure the packet arrival time (Tx) for the minimum TTA frame size (60 bytes),
- Measure the packet transmission time (Rx) for the minimum TTA frame size (60 bytes),
- Measure the packet arrival time (Tx) for the maximum TTA frame size (1514 bytes),
- Measure the packet transmission time (Rx) for the maximum TTA frame size (1514 bytes).

Table 6 shows the values of the performed tests (due to network communication overhead). Based on these data, it is possible to compute the switch performance. For that, it is necessary to compute the time difference (Diff) between the time that the switch received the packet (Tx) with the time that the switch forwarded the packet (Rx) to the respective channel.

(3) SwitchPerformance:Diff=Tx−Rx

The average value for Diff at 60 bytes (0.378 ms) and 1514 bytes (0.372 ms) is approximately the same and, it reflects the consistency and determinism of the central switch in forwarding different frame sizes. In theory, this Diff should be the same as the guard period (0.2 ms) mentioned in Section 5. One of the factors that contributes to the extra overhead is the addition of the extra switch to sniff the communication channel. It is important to mention that all of the values from Table 6 include this overhead. The additional switch takes different times to forward the packets, which are also dependent on the size of the packets. If the theoretical guard period (0.2 ms) from the Diff results (0.378 ms) is removed, the overhead introduced by the additional switch is left. For the remaining calculations, a boundary of 0.1 ms is assumed for the additional Switch Overhead (SO).

Based on the collected data, it is also possible to obtain the time that the transmitted packet takes from one end node to the central switch. Once again, this is possible to compute because all end nodes are in sync with the central switch by the sync beacon packet. From Table 6, the average transmission time (Tx) for 60 bytes is 0.008 ms and 0.222 ms for 1514 bytes. As expected, the end node takes more time to transmit more data. This is due to the time that the network device drivers at the end node take to make the data available to transmit.

The maximum transmission time ($Max_{TT}$) can be calculated with the following equation:

(4) $$Max_{TT}=\left(2\times \left(Frame_{time}-SO\right)+Diff\right)$$

where $Frame_{time}$ is the time that it takes for the message to go from the end node to the central switch and *Diff* the time that the central switch takes to forward the message. In all experiments, it is assumed that the network device drivers’ delay at the receiver end node is the same as the network drivers’ delay at the transmitter node.

As an example, from Table 6, taking the maximum transmission time for 60 bytes (0.115 ms) and with Diff equal to the guard period (0.2 ms), the following $Max_{TT}$ is obtained:

(5) $$Max_{TT}=\left(2\times \left(0.115-0.1\right)\right)+0.2=0.23\;\text{ms}$$

Using the SHA-1 message tag overhead and using the equation mentioned above ($Max_{TT}$), it is possible to evaluate the performance impact on the maximum number of frames per BP by adding extra bytes to ensure secure authentication. Table 7 shows the performance impact values (Note that the $Max_{TT}$ for the Measured results from Table 7 has the additional switch overhead subtracted).

#### 7.1.3. Interpretation of the Results

There is a significant drop in the maximum number of frames per BP between the theoretical and measured values. The main reason is that the theoretical values do not include the time that the device drivers from the end node side take to make available the frame to transmit. The end node network device driver’s time is not deterministic, and it depends on several factors, such as the type of operating system running on it and the amount of data desired to transmit.

Moreover, based on the measured values, there is a small impact on the maximum number of frames per BP by increasing the packet size from 60 to 80 bytes (in this case, using the SHA-1 message tag). The time that it takes to transmit a frame with or without the generated hash tag is approximately the same, and it does not affect the real-time execution of the application example.

### 7.2. Platform B

#### 7.2.1. Physical Measurement Setup

For Platform B, the approach needs to be different. Because the network channel is wireless, therefore it is not feasible, by “sniffing” the packets, to find a common clock base between the nodes. Moreover, since the internal timer of the nodes cannot be used because of the clock skew, the approach relies on using GPIO (General Purpose Input and Output) pins available in the end nodes and connecting them to an oscilloscope. The GPIO pins are toggled to specify an event in the system.

**Figure 9:**
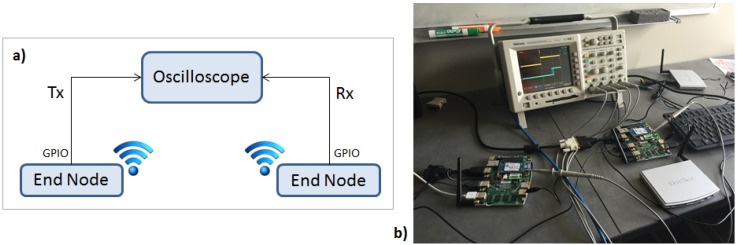
Measurement setup: (**a**) Block diagram; (**b**) Physical setup.

Figure 9 shows the experimental setup. The oscilloscope used is a Tektronix TDS 3054B with 500 MHz of bandwidth and four analog channels.

**Figure 10:**
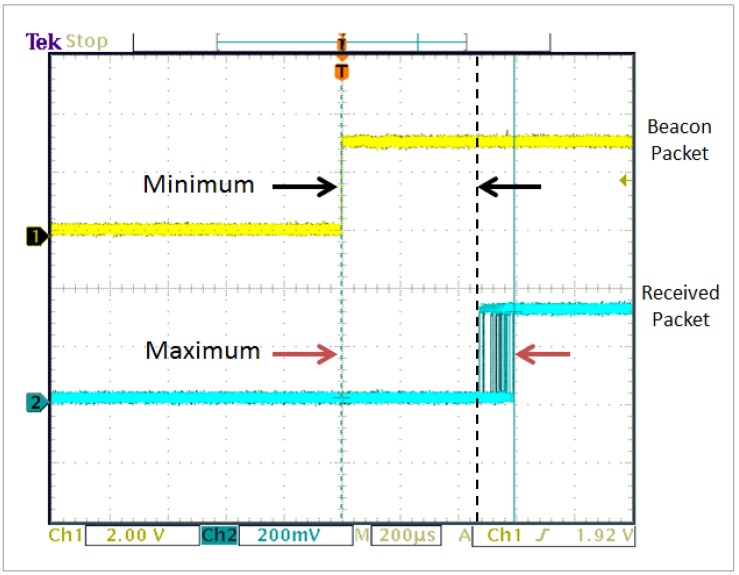
Oscilloscope packet transmission measurement.

One node is assigned as the master node of the TDMA scheduler. The master node broadcasts a beacon packet (Tx) at a predefined interval (10 ms), and it toggles a pin to highlight the event. The remaining nodes toggle a pin upon reception (Rx) of that beacon packet. The packet transmission time is then measured by taking the difference between these two events. Figure 10 shows an oscilloscope measurement (60 bytes plus SHA-1 20 bytes tag = 80 bytes) of the packet propagation time.

#### 7.2.2. Experimental Results

From Figure 10, the measured time (minimum or maximum) is related to the packet transmission time, but it does not include the time that the end node network device driver takes to pass and receive the packet to the physical layer. With this in mind, measurements are made in two parts.

The first part of the experiment involves calculating the time it takes the network device driver to attach the network headers and pass it to the physical layer and vice versa. It is important to note that these times are hardware dependent and may vary for different hardware and operating systems. To calculate these times, the GIPO pins are toggled just before and after the send and receive function. Table 8 presents the different network device driver times for the send function. Since the use of SHA-1 adds 20 bytes of data, the packet size was varied from 60 to 80 bytes and from 1280 to 1300 bytes.

As mentioned in Section 3, all of the software developed is running in the kernel space, and the packet sockets used to receive and send packets are at the device driver level (data link layer: OSI Layer 2). When data are sent through the socket, the data link layer is responsible for several different operations (e.g., queuing or traffic-shaping functions), apart from just handing over the packet to the physical layer. The main function at this level is to schedule the packet to be sent out. For this purpose, Linux uses a queuing mechanism. The packet is added to a queue and sent out into the medium by calling a set of I/O instructions to copy the packet to hardware and start transmitting. After the packet transmission is complete, the device frees the queue. If the transmission fails for any reason, then the packet is re-queued again for processing later. If for some reason the packet transmission could not occur, then the packet transmission is scheduled again in the soft Interrupt Request (IRQ) context. This explains the variability present in the values for the send function (Table 8).

On the other hand, the measured receive function time has a constant value of 1 μs independent of the packet size, due to the high priority IRQ being used for handling incoming data. When data are received from the medium, the card receives the packet, and the packet is transferred directly through DMA to the kernel memory space. After the packet is transferred to the kernel memory, the card interrupts the CPU, to inform about the availability of a new packet. The CPU then transfers the control to the core Interrupt Service Routine (ISR), which will take care of the packet processing. As the processing in the interrupt context should be as low as possible, the ISR initiates the soft IRQ context, which will take the packet processing further.

The second part of the experiment is related to the measurement of the packet transmission time. The results for this case do not include the send function time, but they include the negligible and deterministic receiving function time (described above). Since the use of SHA-1 adds 20 bytes of data, the packet size was varied from 60 to 80 bytes and from 1280 to 1300 bytes. Table 9 presents the minimum and maximum packet transmission time for the respective packet size.

As expected, it takes more time to transmit more data. Once again, the transmission is not deterministic; it varies with the packet size. However, the packet transmission time is relatively the same for an additional 20 bytes.

The maximum transmission time is equal to the packet transmission time plus the send function network device driver time plus the guard period:

(6) $$Max_{TT}=Frame_{time}+GP$$

where,

(7) $$Frame_{time}=Packet\;\;Transmission\;\;Time+Send\;\;Function\;\;Time$$

As an example, from Table 8 and Table 9, taking the maximum time for a packet size of 60 bytes and with a guard period of 0.2 ms, the following $Max_{TT}$ is obtained:

(8) $$Max_{TT}=\left(0.584\;\left(\text{ms}\right)+0.240\;\left(\text{ms}\right)\right)+0.2\;\left(\text{ms}\right)=1.024\;\left(\text{ms}\right)$$

Considering the $Frame_{time}$ with the SHA-1 message tag overhead, it is possible to evaluate the performance impact on the maximum number of frames per BP. Table 10 shows the performance impact.

#### 7.2.3. Interpretation of Results

There is a drop in the maximum number of frames per BP between the theoretical and measured values. One reason is related to the fact that the theoretical values do not take the send function time into consideration. Another problem relates to the nature of WiFi technology that makes throughput hard to predict [20]. External factors, such as radio and/or physical and/or electrical interference, distance between radios, physical obstacles and other wireless networks, can have an impact on the observed maximum throughput. As mentioned in [21], a maximum raw data rate of 54 Mbits/s can typically yield a throughout in the mid-20 Mbits/s, more than 50% of the bandwidth reduction. In order to measure the bandwidth performance for the wireless setup, Iperf (https://iperf.fr/) was used. Iperf is a tool that allows one to measure the maximum TCP and UDP bandwidth performance. The results obtained were in the range of 14 to 20 Mbits/s. Another issue that can contribute to such a difference between the theoretical and measured values is related to the Medium Access Control (MAC) protocol used in the WiFi 802.11 standards. The MAC protocol is not deterministic, because it relies on random times to access the transmission medium to avoid packet collisions.

Similarly to the previous section, there is no impact on the maximum number of frames per BP by increasing the packet size from 60 to 80 bytes (in this case, using the SHA-1 message tag). The time that it takes to transmit a frame with or without the generated hash tag is approximately the same, and it does not affect the real-time execution of the application example.

## 8. Scalability Analysis

This section presents the scalability analysis for the communication overhead. The aim is to present the impact on the $NF_{max}$ evaluation metric, as the number of nodes increases, using the experimental values from Section 7 by scaling the TT networks.

### 8.1. Platform A

For Platform A (star network topology; Section 5.1), the theoretical maximum number of messages per BP is 48 (Table 3). The switch used during the experiments (Section 7.1) has eight ports. Therefore, it is possible to connect up to eight different end nodes to it. If this is the case, then each node can transmit up to six messages per BP. If the application requires connecting more than eight end nodes, it is necessary to increase the number of ports. There are two possible ways to achieve this: use a switch with more ports or connect several switches.

**Figure 11:**
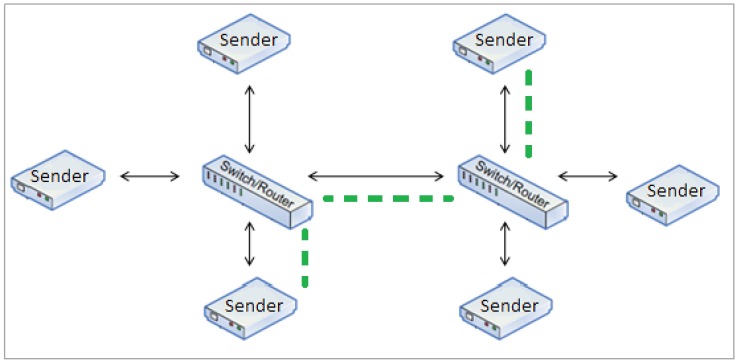
Scalability: Platform A, cascade network topology.

The first solution does not have an impact on the calculation of $NF_{max}$. As long as the switch has enough ports for the required number of end nodes, and the minimum guard period (0.2 ms) used to ensure that physical switch is fast enough to route one frame at its maximum size is the same, the calculation remains the same. However, this becomes an impractical solution, since every time there is a need to add more end nodes, the switch needs to be changed accordingly. The second solution is more practical, and it relies on connecting switches in a cascade topology (Figure 11).

In order to calculate the maximum number of frames per BP, every time a new switch is added, there is an additional guard period that needs to be added to the TDMA frame (Equation (9)).

(9) $$NF_{max}=\frac{BP}{\left(Frame_{time}\right)+\left(GP*N_{Switch}\right)}$$

In order to measure the scalability impact on $NF_{max}$, the $Frame_{time}$ from the experimental values from Section 7.1.2 are used in the equation above. Table 11 shows the respective results. In this case, there is significant impact on $NF_{max}$. By authenticating the messages (60 to 80 bytes), the impact is negligible on $NF_{max}$, and there is no impact on the number of end nodes (# Nodes) that can transmit in the same TDMA frame. On the other hand, after adding the third switch, the number of nodes that can be connected to the switch ports is bigger than the actual number of messages that can be transmitted per BP. The additional guard period starts to take significant space in the TDMA frames, reducing the number of messages that can be transmitted.

### 8.2. Platform B

For Platform B (mesh network topology; Section 5.2), scalability is addressed in a different way. The theoretical maximum number of messages per BP is 47 (Table 5), and the network topology is a mesh configuration (no switch). In this case, each message per BP can be an end node (47 end nodes total). If the application requires connecting more end nodes (fixing BP and GP from Equation (1)), then it is necessary to decrease the $Frame_{time}$, that is increase the $Transmission_{rate}$ from Equation (2) to allow adding more end nodes to the TDMA frame.

## 9. Conclusions

Time-triggered network control systems are a fundamental part of the communication components of current cyber-physical systems (e.g., automotive/planes communications). They allow safe, reliable and fault-tolerant network communications. Nonetheless, secure communication mechanisms need to be incorporated without affecting the overall system stability, and the impact performance of secure messages should be analyzed in existing network communications.

This paper presents an authentication method based on a keyed-Hashed Authentication Code (HMAC) with the aim to enable secure communications between several nodes. The computation and communication performance of the bus communication by adding this level of security on the messages is analyzed on two different platforms (Platform A, wired, and Platform B, wireless networked control systems). For both platforms, the computation overhead for implementing HMAC does not affect the overall system performance. The algorithms are implemented at the operating system kernel level, and the execution time is negligible for the end nodes that are authenticating the messages.

Regarding the communication overhead, it is important to highlight that the packet size will have a direct influence not only on the time that it takes to transmit a packet, but also on the number of frames per BP in the communication scheme for TT networks. For the wired network (Platform A), there is a small impact on the maximum number of frames per base period (BP) by including secure messages. On the other hand, for the wireless network (Platform B), there is no impact on the maximum number of frames per BP by including secure messages.

Overall, in this case study, it is feasible to implement a secure communication mechanism (HMAC) for TT networked control systems without interfering with the existing computation and communication controller execution times.

Both HMAC and digital signatures enable message integrity and authentication. Moreover, both are capable of providing identical levels of security. Digital signatures are normally slower than HMAC methods, but digital signatures enable the non-repudiation property, that is a node that transmitted some information cannot at a later time deny having signed it. As future work, it will be interesting to measure the computational and communication overhead by implementing a digital signature approach and comparing the respective results with the HMAC approach.

## Figures and Tables

**Table 1:** Notation and terminology.

Notation	Terminology
BP	Base Period
Diff	Time Difference (Switch Performance)
ECUs	Electronic Control Units
GP	Guard Period
HMAC	Hash-Based Message Authentication
MaxTT	Maximum Transmission Time
NFmax	Maximum Number of Frames
Nswitch	Number of Switches
Rx	Packet Transmission Time
SO	Switch Overhead
TDMA	Time-Division Multiple Access
TTA	Time-Triggered Architectures
Tx	Packet Arrival Time
WS	Additional Switch (Wireshark)
# Nodes	Number of End Nodes

**Table 2:** Platform A: experiment parameters.

Parameter	Description	
$Transmission_{Rate}$	Transmission Rate	100 Mbits/s
GP	Guard Period	0.2 ms
$Packet_{Size}$	Packet Size	Min: 60 bytes Max: 1514 bytes
BP	Base Period	10 ms
Tag	SHA-1 Hash Tag Size	20 bytes

**Table 3:** Platform A: theoretical results (with BP = 10 ms).

	GP = 0		GP = 0.2 ms	
	60 bytes	80 bytes	60 bytes	80 bytes
$NF_{max}$	2083	1563	48	48
$Frame_{time}$ (ms)	0.0048	0.0064	0.2048	0.2064

**Table 4:** Platform B: experiment parameters.

Parameter	Description	
$Transmission_{Rate}$	Transmission Rate	54 Mbits/s
GP	Guard Period	0.2 ms
$Packet_{Size}$	Packet Size	Min: 60 bytes Max: 1300 bytes
BP	Base Period	10 ms
Tag	SHA-1 Hash Tag Size	20 bytes

**Table 5:** Platform B: theoretical results (with BP = 10 ms).

	GP = 0		GP = 0.2 ms	
	60 bytes	80 bytes	60 bytes	80 bytes
$NF_{max}$	1125	843	47	47
$Frame_{time}$ (ms)	0.0089	0.0119	0.2089	0.2119

**Table 6:** Central switch performance measured values. Diff, time difference.

	60 bytes			1514 bytes		
	Min	Average	Max	Min	Average	Max
Tx (ms)	0	0.0080	0.1150	0.1100	0.2220	0.3470
Rx (ms)	0.2740	0.3860	0.6730	0.4940	0.5940	0.8260
Diff (ms)	0.2740	0.3780	0.5580	0.3840	0.3720	0.4790

**Table 7:** Platform A: performance impact results.

	Theoretical		Measured	
	60 bytes	80 bytes	60 bytes	80 bytes
$NF_{max}$	48	48	43	33
$Frame_{time}$ (ms)	0.0048	0.0064	0.1150	0.1500
$Max_{TT}$ (ms)	0.2096	0.2128	0.2300	0.3000

**Table 8:** Send function: network device driver time.

Packet Size (bytes)	Min (μs)	Max (μs)
60	208	240
80	208	240
1280	240	320
1300	240	320

**Table 9:** Packet transmission time.

Packet Size (bytes)	Min (μs)	Max (μs)
60	432	584
80	432	592
1280	696	888
1300	700	896

**Table 10:** Platform B: performance impact results.

	Theoretical		Measured	
	60 bytes	80 bytes	60 bytes	80 bytes
$NF_{max}$	47	47	9	9
$Max_{TT}$ (ms)	0.2089	0.2119	1.024	1.032

**Table 11:** Platform A: scalability practical results (with BP = 10 ms).

GP = 0.2 ms					
60 bytes			80 bytes		
$N_{\mathrm{switch}}$	${\mathrm{NF}}_{\max}$	# Nodes	$N_{\mathrm{switch}}$	${\mathrm{NF}}_{\max}$	# Nodes
1	31	8	1	28	8
2	19	14	2	18	14
3	13	20	3	13	20
4	10	26	4	10	26
5	8	32	5	8	32

## References

[1] Suri N., Walter C.J., Hugue M.M. (1994). Advances in Ultra-Dependable Distributed Systems.

[2] Kopetz H., Bauer G. (2003). The time-triggered architecture. IEEE Proc..

[3] Sztipanovits J., Koutsoukos X., Karsai G., Kottenstette N., Antsaklis P., Gupta V., Goodwine B., Baras J., Wang S. (2012). Toward a Science of Cyber-Physical System Integration. IEEE. Proc. Spec. Issue Cyber-Phys. Syst..

[4] Navet N., Song Y., Simonot-Lion F., Wilwert C. (2005). Trends in automotive communication systems. IEEE Proc..

[5] Lee E.A. Cyber Physical Systems: Design Challenges. Proceedings of the 11th IEEE International Symposium on Object Oriented Real-Time Distributed Computing (ISORC).

[6] Nolte T., Hansson H., Bello L. Automotive Communications—Past, Current and Future. Proceedings of the 10th IEEE Conference on Emerging Technologies and Factory Automation (ETFA).

[7] Kopetz H. The time-triggered model of computation. Proceedings of the 19th IEEE Real-Time Systems Symposium.

[8] Steiner W. Candidate Security Solutions for TTEthernet. Proceedings of IEEE/AIAA 32nd Digital Avionics Systems Conference (DASC).

[9] Song J., Han S., Mok A.K., Chen D., Lucas M., Nixon M. WirelessHART: Applying Wireless Technology in Real-Time Industrial Process Control. Proceedings of the IEEE Real-Time and Embedded Technology and Applications Symposium.

[10] Lennvall T., Svensson S. A Comparison of WirelessHART and ZigBee for Industrial Applications. Proceedings of the IEEE International Workshop Factory Communication Systems.

[11] National Research Council (US) (2012). Committee on Electronic Vehicle Controls and Unintended Acceleration. The Safety Promise and Challenge of Automotive Electronics: Insights from Unintended Acceleration.

[12] Koscher K., Czeskis A., Roesner F., Patel S., Kohno T., Checkoway S., McCoy D., Kantor B., Anderson D., Shacham H. Experimental security analysis of a modern automobile. Proceedings of the IEEE Symposium on Security and Privacy (SP).

[13] Wolf M., Weimerskirch A., Paar C. Security in Automotive Bus Systems. Proceedings of the Workshop on Embedded Security in Cars (ESCAR).

[14] Kargl F., Papadimitratos P., Buttyan L., Müter M., Schoch E., Wiedersheim B., Thong T., Calandriello G., Held A., Kung A. (2008). Secure Vehicular Communication Systems: Implementation, Performance, and Research Challenges. IEEE Commun. Mag. Top. Autom. Netw..

[15] Patsakis C., Dellios K., Bouroche M. (2014). Towards a distributed secure in-vehicle communication architecture for modern vehicles. Comput. Secur..

[16] Wasicek A., El–Salloum C., Kopetz H. Authentication in Time Triggered Systems using Time delayed Release of Keys. Proceedings of the 14th IEEE International Symposium on Object/Component/Service-Oriented Real-Time Distributed Computing.

[17] Stallings W. (2010). Cryptography and Network Security: Principles and Practices.

[18] Gilbert H., Handschuh H. (2004). Security Analysis of SHA-256 and Sisters. Selected Areas in Cryptography.

[19] Zhang Z., Eyisi E., Koutsoukos X., Porter J., Karsai G., Sztipanovits J. (2014). A co-simulation framework for design of time-triggered automotive cyber physical systems. Simul. Model. Pract. Theory.

[20] Lee H.C., Guo S. (2012). A MAC throughput in the wireless LAN. Advanced Wireless LAN.

[21] Tektronix Wi-Fi: Overview of the 802.11 Physical Layer and Transmitter Measurements. www.tektronix.com/wifi.

